# Prehaustorial local resistance to coffee leaf rust in a Mexican cultivar involves expression of salicylic acid-responsive genes

**DOI:** 10.7717/peerj.8345

**Published:** 2020-01-22

**Authors:** Edgar Couttolenc-Brenis, Gloria L. Carrión, Luc Villain, Fernando Ortega-Escalona, Daniel Ramírez-Martínez, Martín Mata-Rosas, Alfonso Méndez-Bravo

**Affiliations:** 1 Red de Manejo Biotecnológico de Recursos, Instituto de Ecología, A.C., Xalapa, Veracruz, México; 2 Red de Biodiversidad y Sistemática de Hongos, Instituto de Ecología, A.C., Xalapa, Veracruz, México; 3 La Recherche Agronomique pour le Développement, UMR, RPB, CIRAD, Montpellier, France; 4 Red de Ecología Funcional, Instituto de Ecología, A.C., Xalapa, Veracruz, México; 5 Escuela Nacional de Estudios Superiores Unidad Morelia, Universidad Nacional Autónoma de México, Morelia, Michoacán, México; 6 CONACYT-Escuela Nacional de Estudios Superiores Unidad Morelia, Laboratorio Nacional de Análisis y Síntesis Ecológica, Universidad Nacional Autónoma de México, Morelia, Michoacán, México; 7 Current affiliation: Instituto Nacional de Investigaciones Forestales Agrìcolas y Pecuarias, C.E. Cotaxtla, Veracruz, México

**Keywords:** Coffea arabica, Defense gene-expression, Leaf rust, Salicylic acid, SAR

## Abstract

**Background:**

In Mexico, coffee leaf rust (CLR) is the main disease that affects the Arabica coffee crop. In this study, the local response of two Mexican cultivars of *Coffea arabica* (Oro Azteca and Garnica) in the early stages of *Hemileia vastatrix* infection was evaluated.

**Methods:**

We quantified the development of fungal structures in locally-infected leaf disks from both cultivars, using qRT-PCR to measure the relative expression of two pathogenesis recognition genes (*CaNDR1* and *CaNBS-LRR*) and three genes associated with the salicylic acid (SA)-related pathway (*CaNPR1*, *CaPR1,* and *CaPR5*).

**Results:**

Resistance of the cv. Oro Azteca was significantly higher than that of the cv. Garnica, with 8.2% and 53.3% haustorial detection, respectively. In addition, the *non-race specific disease resistance gene* (*CaNDR1*), a key gene for the pathogen recognition, as well as the genes associated with SA, *CaNPR1*, *CaPR1,* and *CaPR5*, presented an increased expression in response to infection by *H. vastatrix* in cv. Oro Azteca if comparing with cv. Garnica. Our results suggest that Oro Azteca’s defense mechanisms could involve early recognition of CLR by NDR1 and the subsequent activation of the SA signaling pathway.

## Introduction

Coffee leaf rust (CLR) is one of the most destructive diseases of the Arabica crop worldwide. The causal agent is the biotrophic fungus *Hemileia vastatrix* Berk. et Br. (Berkeley & Broome, 1869 in, Diola et al., 2013; Zambolim, 2016; Talhinhas et al., 2017), a pathogen specific to the *Coffea* genus (Thirumalachar & Narasimhan, 1947). In Mexico, about 50% of coffee plantations were affected by CLR during 2017 (PEVEF-Cafeto, 2017).

Although the production of rust-resistant varieties of *Coffea* has been promoted by generating hybrids, most of the hybrids have been derived from Híbrido de Timor (a spontaneous interspecific cross between *Coffea arabica * and *C. canephora*) (reviewed by Talhinhas et al., 2017). Furthermore, monogenic and oligogenic resistance, conferred by the system gene-for-gene (Flor, 1971), has been increasingly overcome by virulence factors of the pathogen (Rodrigues Jr, Gonçalves & Várzea, 2004; Cabral et al., 2009; Fernandez et al., 2012; Maia et al., 2013; Zambolim, 2016). CLR resistance is ruled by at least nine major dominant genes (*SH1*-*SH9*) that recognize pathogen virulence genes (*V1*-*V9*). Combinations of virulence genes define the physiological races of *H. vastatrix* (Gichuru et al., 2012). Fifty distinct races have been identified worldwide (Várzea & Marques, 2005). The promotion of tolerance to the infectious process, therefore, through multigenic co-regulated pathways at the early stages of the pathogenic interaction is a useful tool to induce the expression of defensive mechanisms against the disease.

The response of plants to the attack of a pathogen begins with the recognition of the pathogen. This recognition can occur extracellularly when pathogen-associated molecular patterns (PAMPs) are recognized by pattern recognition receptors (PRRs), activating the pathogen-triggered immunity (PTI) mechanism (Jones & Dangl, 2006; Dodds & Rathjen, 2010). A second mode of immunity is based on the identification of molecules of adapted pathogens (effectors) at the intercellular level. These activate the effector-triggered immunity (ETI) mechanism. Although both defense mechanisms generate common responses, including an oxidative burst, hormonal changes, and transcriptional reprogramming (Dodds & Rathjen, 2010), the activated immune responses of ETI are more prolonged and robust than those of PTI (Katagiri & Tsuda, 2010). PTI is effective against non-adapted pathogens (non-host resistance), ETI against adapted pathogens (Katagiri & Tsuda, 2010). Among the recognition genes that have been identified in coffee, *NDR1* is related to the early activation of both defense mechanisms (Knepper, Savory & Day, 2011b). Its function in PTI has been described as indispensable for the activation of the mitogen-activated protein kinase (MAPK) signaling pathway (Knepper, Savory & Day, 2011b). MAPK signaling during ETI is related to the activation of some of the genes that encode the coiled-coil–nucleotide-binding site–leucine-rich repeat (CC-NB-LRR) protein family (Dodds & Rathjen, 2010; Knepper & Day, 2010). Both PTI and ETI activate plant defensive responses related to the salicylic acid (SA) and jasmonic acid (JA)/ ethylene (ET) signaling pathways. The SA signaling pathway is the most important in the immune response to biotrophic pathogens such as *H. vastatrix*.

The coffee-*H. vastatrix* interaction initiates with the adhesion of urediniospores to the abaxial side of the leaves. Once established, the spores germinate and the appressoria are formed. Afterward, the fungus penetrates through the stomata, forming a penetration hypha. The stomata chambers are invaded by haustoria and intracellular mycelium, beginning the colonization of the mesophyll cells (Coutinho, Rijkenberg & Van Asch, 1993; Zambolim, 2016; Talhinhas et al., 2017). During these first infection stages, recognition of the fungus by the plant induces defense mechanisms that lead resistant plants to have an incompatible interaction through hypersensitive response (HR) at the appressorial or pre-haustorial stage (Diola et al., 2013; Florez et al., 2017). The HR is mostly regulated by the salicylic acid-responsive (SA) signaling pathway and is associated with the local production of oxygen reactive and phenolic compounds, callose deposition, and cell wall lignification (Silva et al., 2002; Florez et al., 2017).

The expression level of genes involved in resistance responses against CLR has been characterized in several incompatible interactions at early stages. Among them are: the recognition encoding genes *CaRLK*, *CaR111*, *CaNBS-LRR*, and *CaNDR1* (Naruzaka et al., 2013a; Naruzaka et al., 2013b; Selote et al., 2013; Bao et al., 2016); the SA-associated genes *CaNPR1*, *CaPR1*, *CaPR5* (Kim & Hwang, 2014; Diniz et al., 2017; Jain & Khurana, 2018); the transcriptional regulators type-AP2 and *WRKY* families (Fernandez et al., 2004; Fernandez et al., 2012; Ganesh et al., 2006; Ramiro et al., 2010); the genes encoding oxidative enzymes (lipoxygenases, peroxidases, and superoxide dismutase); phenylalanine ammonia lyase, chalcone synthase, chitinases and glucanases (Fernandez et al., 2004; Fernandez et al., 2012; Ganesh et al., 2006; Diniz et al., 2012; Ahuja, Kissen & Bones, 2012; Shanshan et al., 2017; Yahyaa et al., 2017; Jaggi, 2018; Florez et al., 2017); and several genes associated with the production of flavonoids (*CaPAL* and *CaCHS*) and signaling protein kinases (*CaMAPK2*, *CaMEK* and *CaCDPK*) (Ahuja, Kissen & Bones, 2012; Shanshan et al., 2017; Yahyaa et al., 2017; Jaggi, 2018). These genetic backgrounds constitute an important stock of marker genes to distinguish between resistant and susceptible cultivars. The expression profiles of these genes allow us to understand the defense mechanisms involved in resistance to CLR.

As a result of the increasing damage caused by CLR in Mexico, the growing of resistant cultivars has been promoted. Among these cultivars, Oro Azteca has been the most planted in Mexican fields (Juárez-Bravo et al., 2018). This variety is a “Catimor”, which has been defined as a group of introgressed Arabica varieties originated from the cross of Híbrido de Timor CIFC 832/1 with cv. Caturra CIFC 19/1 (Montagnon, Marraccini & Bertrand, 2012; World Coffee Research, 2018; Luna et al., 2019) and developed by the National Institute of Forestry and Agriculture Research (INIFAP). Information about the response of this variety to CLR infection, however, is scarce.

Therefore, the main objectives of our study were to evaluate the local response of two Mexican cultivars of *C. arabica* at early stages of infection (germination, appressoria and haustoria formation) by *H. vastatrix* under controlled conditions in order to quantify the expression levels of some defense-related genes as well as to describe the possible mechanism involved in the resistance reaction against CLR. The selected cultivars were the Catimor Oro Azteca and cv. Garnica, developed by the Mexican Coffee Institute (IMECAFE) from the cross of cv. Caturra Amarillo with cv. Mundo Novo (Rivera & Villareal, 2015).

## Materials & Methods

### Plant material and fungal inoculation

**Figure 1:**
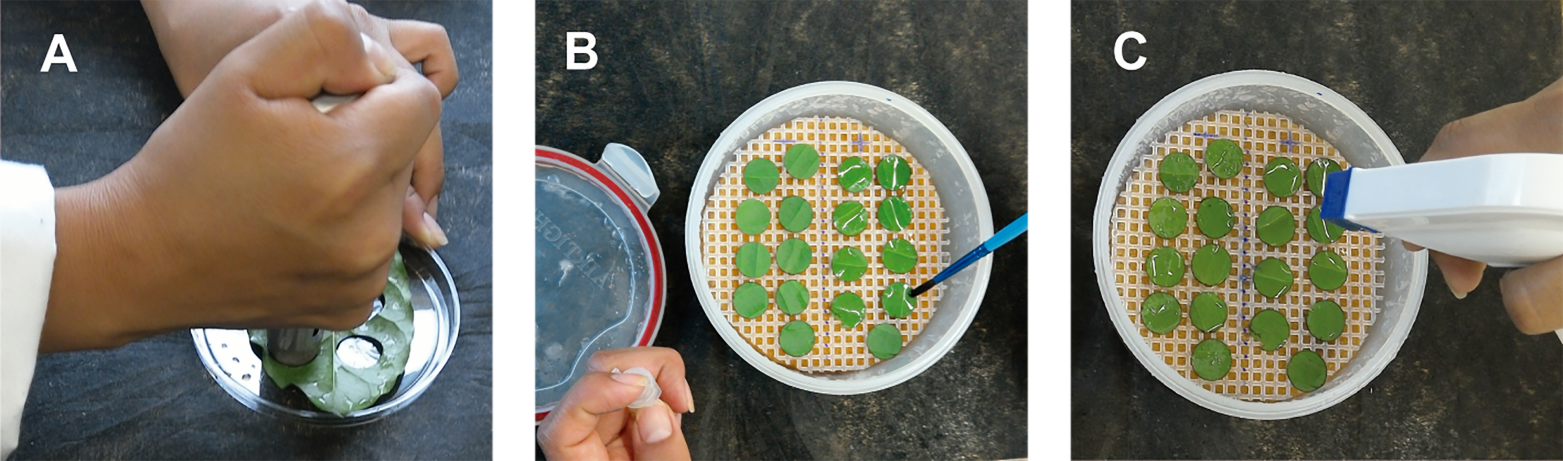
Scheme showing the preparation of coffee leaf disks and the inoculation of *Hemileia vastatrix* uredospores. (A) Cutting of leaf disks with a cork borer; (B) uredospore spreading over the abaxial surface of the disks with a camel hairbrush, and (C) spraying the control (non-inoculated) or inoculated leaf disks with distilled water to keep a high relative humidity.

To evaluate the local response of cv. Oro Azteca to *H. vastatrix* infection, we established an experiment with inoculated and uninoculated leaf disks. Cv. Oro Azteca and the CLR-susceptible cv. Garnica plants were grown under greenhouse conditions. We selected ten plants from each cultivar with at least four pairs of leaves. One leaf from each plant was collected from the second pair of leaves. From each selected leaf, we cut 12 disks with a 1.8 cm diameter cork borer. In total 120 leaf disks from each cultivar were employed for the analyses. The disks were placed in airtight plastic containers over a moistened foam and plastic mesh. We obtained the uredospores of *H. vastatrix* by collecting leaves from infested coffee plantations at the monitoring area of the phytosanitary epidemiological vigilance program of the National Department of Agriculture (SEDAR) in Huatusco, Veracruz, Mexico. We isolated the uredospores from the leaves in the laboratory with a stereoscope and placed them in 2 ml microcentrifuge tubes. Each tube was filled with a solution of 0.01% Tween® 80 to obtain a final concentration of 1.5 × 10^5^ uredospores ml^−1^. We inoculated leaf disks with a camel hair brush to spread the uredospores over the abaxial surface as previously reported by Cabral et al. (2009). The containers were incubated in the dark for 48 h, at 24 ± 1 °C and a high relative humidity to allow the uredospore germination. The leaf disks were then incubated in normal laboratory light conditions (Fig. 1).

### Evaluation of fungal growth

To characterize the response of cv. Oro Azteca and cv. Garnica to CLR, we determined the number of fungal structures (percent of uredospore germination and the formation of appressoria and haustoria) in vegetal tissue from inoculated leaf disks. To ensure that we were able to evaluate the response of cultivars once the complete local infective process has been completed, the record of developed fungal structures was performed at 24 and 120 hai following the method described by Silva, Rijo & Rodrigues Jr (1985). The leaf disks were covered with transparent nail polish on the lower surface and 24 h later the nail polish was removed with tweezers to obtain a leaf print. Three leaf prints per cultivar were stained and mounted with blue lactophenol. The leaf prints were observed at a 100× magnification with a Nikon Eclipse E600 light microscope; we scored total uredospores, germinated uredospores, and appressoria. To evaluate the formation of haustoria, we fixed leaf disk (samples) with FAA (10% formaldehyde, 50% ethanol, 5% acetic acid and 35% distilled water) for 24 h. Samples were dehydrated using a graded series of terbutilic alcohol and infiltrated with paraffin at 60 °C (Pratt & Wetmore, 1951; Li et al., 2007; Huang & Yeung, 2015; Stasolla & Yeung, 2015). We fixed leaf disks (samples) with a microtome Leica Biosystems RM 2125 RTS and mounted them on slides; deparaffined using Xylene, decolorated with 70% ethanol warmed to 70 °C and lactophenol, and finally stained with blue lactophenol and mounted in polyvinyl alcohol (PVA). We examined cross sections under the Nikon Eclipse E600 light microscope at a 40x magnification to determine the percentage of haustoria formed per infection point. We observed 150 infection points. The data of germination, appressoria, and haustoria were statistically contrasted with a T-Student test by Infostat® software.

Additionally, the uredospore morphology was analyzed by scanning electron microscopy (SEM) in a JEOL JSM-IT300 microscope. At 24 hai, the leaf disks were fixed in a 4% glutaraldehyde solution for 24 h and washed with phosphate buffer (pH = 7.2). Samples were then dehydrated in a gradient of ethanol (70%, 80%, 90% and absolute ethanol) and subsequently dried with liquid CO_2_ for 15 min in a Toussimis Autosamdri®-815, Series A, incorporated to aluminum stubs and sputter coated with 10-nm gold layer using Denton Desk V sputter coater. The scanning was performed with an accelerating voltage of 20 kV.

### Selection of defense-related genes

To study the initial response of Oro Azteca to CLR infection, we evaluated the relative expression of the non-race pathogen recognition gene *CaNDR1b* (Fernandez et al., 2004; Ganesh et al., 2006; Cacas et al., 2011) and the leucine-reach domain recognition gene *CaNBS-LRR* (Diola et al., 2013; Florez et al., 2017). In addition to these recognition genes, we analyzed the *CaNPR1*, *CaPR1*, and *CaPR5* genes associated with the Salicylic Acid (SA) pathway. The housekeeping gene *CaUbiE2* was used as the internal control (Ganesh et al., 2006; Cruz et al., 2009; Ramiro et al., 2009; Diniz et al., 2012; Borges, Tsai & Caldas, 2012; Diola et al., 2013). To evaluate the growth of CLR in leaf disk tissues, we compared the relative expression of the *H. vastatrix Hv40-Rib* (Vieira et al., 2011) gene with respect to the constitutive gene *CaUbiE2*, as it has been performed with other pathogen models (Eshraghi et al., 2011). We designed the specific oligonucleotides with software Primer3Plus® (Untergasser et al., 2007), (Table 1) by using the National Center for Biotechnology (NCBI) database. Primers were synthesized by the T4Oligo Lab in Irapuato, Mexico.

**Table 1:** Designed primers for the amplification of *CaPR1*, *CaPR5*, *CaNBS-LRR*, *CaNDR1b*, *CaNPR1*, *CaUbiE2* and *Hv40s-Rib* genes used in the expression analyses by qRT-PCR.

**Gen**	**Primer**	**Amplification efficiency**
*CaPR1*	F5′-CAGGAATGCGGGCATTATAC-3′ R5′-CAATCGCATGGGTTTGATAA-3′	0.9729
*CaPR5*	F5′-CTGCCTGAGTTGCAGCAATA-3′ R5′-TTTCCCTTGTTGATGGCTTC-3′	0.9420
*CaNBS-LRR*	F5′-CCAAAAACTTTGGGTTGGTG-3′ R5′-TCCATTGCATTCTCATCTG-3′	0.8887
*CaNDR1b*	F5′-CTTACAGGGCGGTGTCAAAT-3′ R5′-TACCACTAGCCCAGGACAGC-3′	0.9357
*CaNPR1*	F5′-GACGCTGCAGTGAAGAAAC-3′ R5′-TGATAGCTTCCCAGGCATCT-3′	0.8913
*CaUbiE2*	F5′-CCATTTAAACCCCCAAAGGT-3′ R5′-GGTCCAGCTTCGAGCAGTAG-3′	1.0394
*Hv40s-Rib*	F5′-ATGCTAGCACCGCTCTTGAT-3′ R5′-ATCGAGCTTCACTTGCTGGT-3′	–

### RNA extraction

To perform the extraction of RNA, we took 10 inoculated and 10 non-inoculated leaf disks from each cultivar at 0, 24 and 72 hai. The disks were macerated in liquid nitrogen to perform the RNA extraction with the RNeasy kit (Quiagen), according to the manufacturer’s instructions.

### qRT-PCR: Synthesis of cDNA

The first strand of the cDNA was synthesized from 30 µg of total RNA. Each reaction mixture contained: 1.0 µg/ml total RNA, first chain solution 10 × 25 mM MgCl_2_, 10 mM dNTPs, 40 units/ml RNasin Inh, 0.5 mg/ml oligo (dT) and 25 units/ml SuperScript III reverse transcriptase (Invitrogen®). The amplification conditions were 10 min at 70 °C followed by two hours at 42 °C for the synthesis of the second chain.

### qRT-PCR

Each reaction was performed with 3 µl cDNA, 1 × of reaction mixture (20 µl) “SYBR Green PCR Master Mix (Applied Biosystems)” and 5 pmol of each primer. Amplification conditions were: 94 °C for 10 min, 40 cycles at 94 °C for 30 s, 60 °C for 30 s and 72 °C for 40 s. The qRT-PCR amplifications were performed with a thermal cycler 7500 Fast Real-Time PCR System (Applied Biosystems) (Cruz et al., 2009; Ramiro et al., 2009; Borges, Tsai & Caldas, 2012). Four independent replicates were obtained with a standard error less than 0.1 for each sample. Each expression value is the average of these replicas. Calculations were performed with the 7500 Software v2.0.1 (Applied Biosystems). The amplification efficiency for each set of oligonucleotides was determined by performing dilution series (1: 5). The specificity of amplification was calculated by means of dissociation curves, obtaining the fluorescence values ΔΔ between 65 °C and 95 °C. On average, the amplification curves were quantified at cycle 15 of each sample.

### Relative quantification of gene expression

The results of qRT-PCR are based on the detection and quantification of fluorescent markers (Cy3 and Cy5) throughout the PCR reaction (Walker, 2002). Then, it is necessary to determine the threshold value of the cycle (Ct), identifying the amplification cycle in which the intensity of the emission of the fluorescent marker rises exponentially during the amplification reaction. The relative quantification calculations were obtained by adjusting for differences in PCR efficiency between the defense-related genes and the average values and efficiency of the *CaUbi2E* gene as the internal reference, according to the model proposed by Pfaffl (2001) and discussed in Hellemans et al. (2007):

\begin{eqnarray*}QR= \frac{{E}_{Target}^{\Delta Ct(control-inoculated)}}{{E}_{ref}^{\Delta Ct(control-inoculated)}} \end{eqnarray*}

To determine if there were differences between the cultivars, an analysis of the variance and the Tukey test were made with the Infostat® program.

## Results

### Evaluation of fungal growth

**Figure 2:**
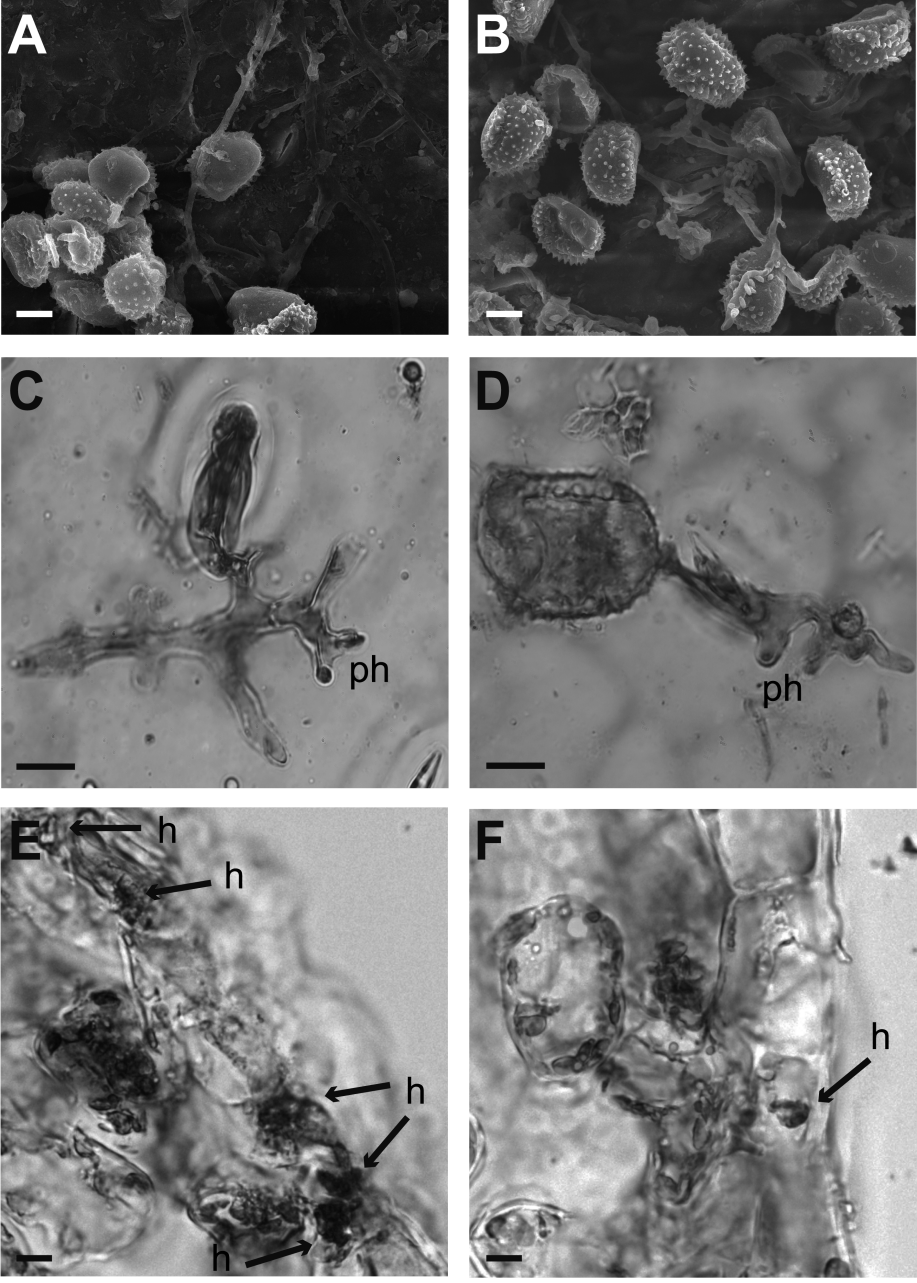
Cytological analysis of inoculated leaf disks. Samples were prepared for their analysis at 24 and 120 hours after inoculation. (A–B) Garnica and Oro Azteca leaf disks surfaces analyzed by Scanning Electron Microscopy. Several uredospores are shown forming the appressorium around a stoma at 24 hai. (C–F) Optical microscope analyses of leaf disks stained with lactophenol blue. (C) Representative stained print of cv. Garnica leaf disk, view of an appressorium on the stoma and a penetration hypha (ph); the remains of the spore were detached from the leaf; (D) print of a representative preparation of leaf disks cv. Oro Azteca; view of one germinated spore of *H. vastatrix*, the appressorium on the stoma and penetration hypha (ph). (E) Presence of fungal tissue in inoculated Garnica leaf disk by analyzing cross sections. Arrows indicate invaded guard cells and haustoria (h). (F) Cross section of one inoculated Oro Azteca leaf disk; arrow points out the haustorium (h). The scale bar means 10 µm.

The percentage of spore germination was similar (*p* = 0.5091) in both varieties, 52.00% in Garnica tissue and 48.80% in Oro Azteca at 24 hai, as was the appressoria number (*p* = 0.2977) (Table 2). The same trend was observed at 120 hai, with 40.14% uredospore germination in Garnica and 37.91% in Oro Azteca leaf disks (*p* = 0.6645), neither the percentage of appressorial detection was statistically different (*p* = 0.2332). In contrast, the percentage of infection points with haustoria was significantly different (*p* < 0.0001). Out of the150 infection points observed for each cultivar, Garnica presented haustoria in 53.33%, Oro Azteca in 8.22% (Table 2; Fig. 2).

**Figure 3:**
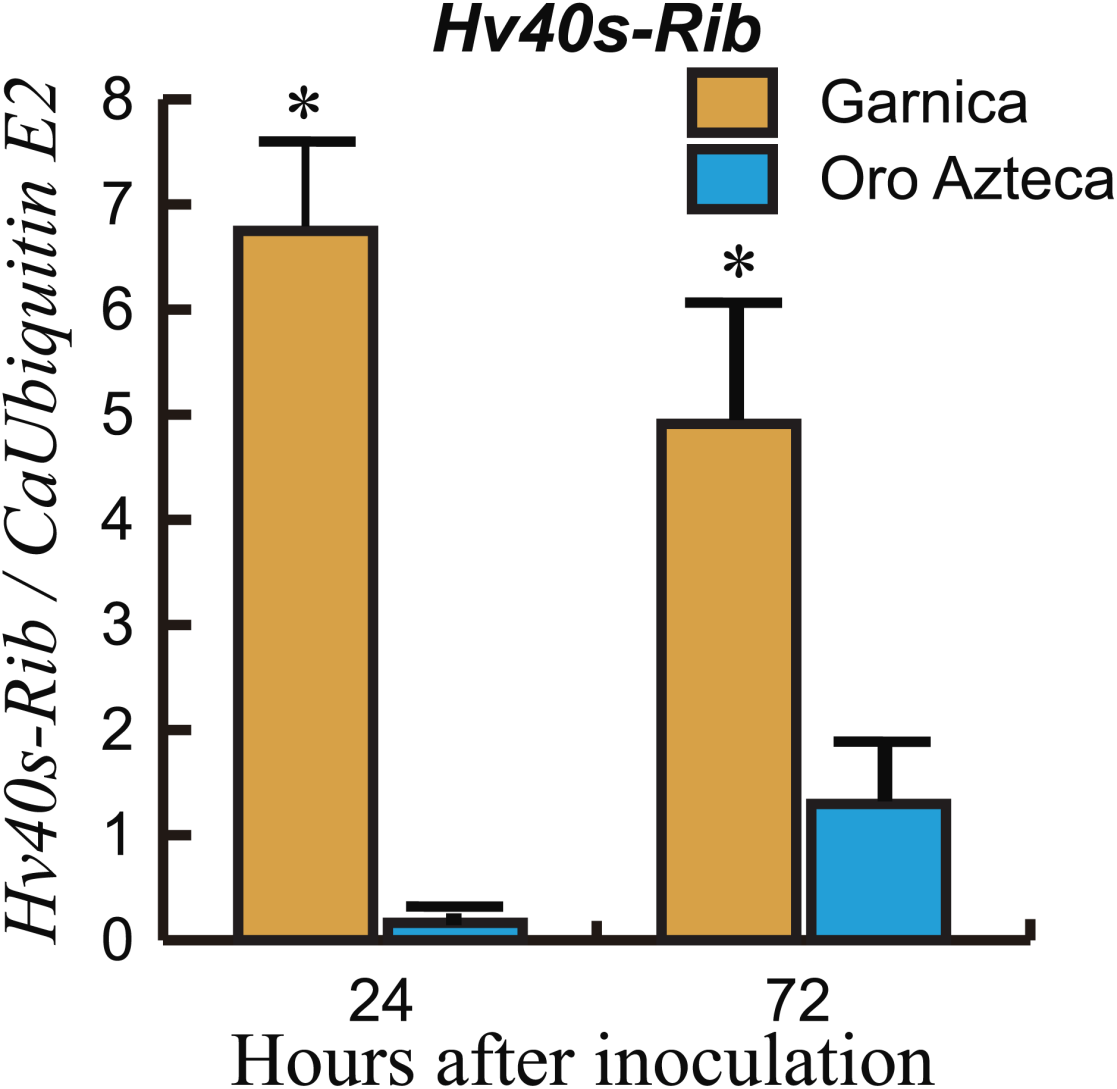
Growth of *H. vastarix* on inoculated leaf disks. Data show the qPCR amplification of *Hv40s-Rib* expression relative to *CaUbiE2* at 24 and 72 hours after inoculation (hai). The ANOVA analysis showed significant differences both at 24 hai (*p* < 0.0002, 7 d.f.) and 72 hai (*p* < 0.0287, 7 d.f). Asterisks indicate differences between the average values of the relative expression (Tukey test, *p* < 0.05).

To corroborate the resistance response in Oro Azteca leaf disks, and to correlate molecular data with fungal colonization, we quantified the expression of the *H. vastatrix Hv40s-Rib* gene relative to the *C. arabica CaUbiE2* gene at 24 and 72 hai. Results indicated a higher proliferation of fungal tissues in Garnica leaf disks than in those of Oro Azteca. The expression level of the endogenous *H. vastatrix Hv40s-Rib* gene was eight-fold and four-fold higher in cv. Garnica as compared with cv. Oro Azteca, at 24 and 72 hai, respectively (Fig. 3).

### Expression of plant pathogen-recognition genes

**Figure 4:**
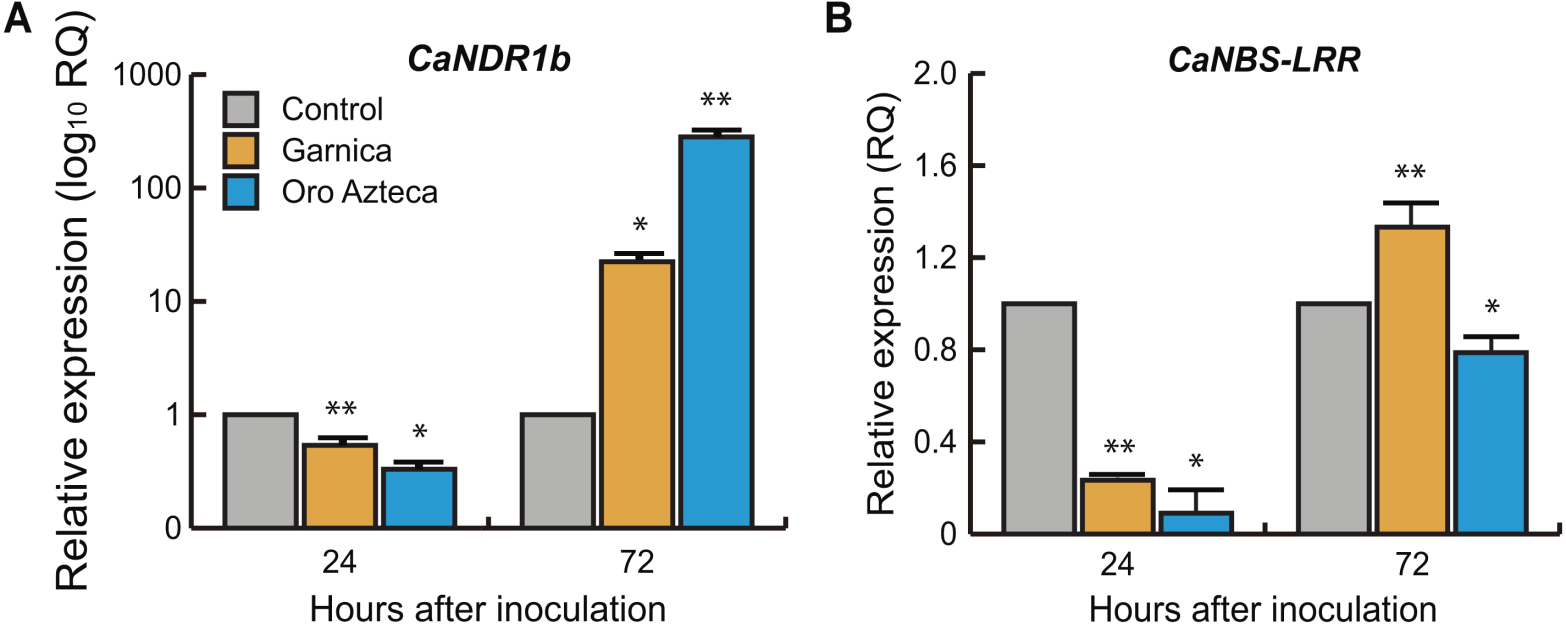
Relative expression of pathogen recognition genes. (A) *CaNDR1b* and (B) *CaNBS-LRR* in Garnica and Oro Azteca infected leaf disks at 24 and 72 hours after inoculation (hai); according to Tukey test (*p* < 0.05), one asterisk indicates differences between the control and the inoculated leaf disk of both cultivars, two asterisks indicate differences with control and between cultivars.

We evaluated the relative expression of the non-race pathogen recognition gene *CaNDR1b* and the leucine-reach domain recognition gene *CaNBS-LRR*. The *CaNDR1b* gene was differentially expressed at 24 hai (*p* < 0.0001, 7 df) and at 72 hai (*p* < 0.0001, 7 df) between cultivars Oro Azteca and Garnica. In both cultivars, the relative expression values were higher with respect to the control (non-inoculated leaf disks) up to 72 hai, and higher in Oro Azteca than in cv. Garnica (Fig. 4A). In the case of *CaNBS*-LRR, the values of relative expression were significant between cultivars (*p* < 0.0.0001, 7 df) at 24 hai and *p* < 0.0002, 7df at 72 hai; cv. Garnica had higher expression with respect to both the control and to cv. Oro Azteca at 72 hai (Fig. 4B).

**Table 2:** Percentage of uredospores germination, appressoria and haustoria detection at 24 and 120 hours after *H. vastatrix* inoculation on leaf disks of *C. arabica* cv. Garnica and cv. Oro Azteca.

Observed structure	cv. Garnica (%)		cv. Oro Azteca (%)		n per cultivar		Degrees of freedom (df)		*p*-value	
	24 hai	120 hai	24 hai	120 hai	24 hai	120 hai	24 hai	120 hai	24 hai	120 hai
Uredospores	52.00 ± 3.58	40.14 ± 2.44	48.80 ± 2.94	37.91 ± 4.10	125	56	8	4	0.5091	0.6645
Appressoria	51.29 ± 5.62	51.85 ± 1.85	43.70 ± 3.85	47.61 ± 2.38	125	56	8	4	0.2977	0.2332
Haustoria^a^	n.a.	53.33 ± 3.11a	n.a.	8.22 ± 1.48b	n.a.	150	n.a.	213	n.a.	<0.0001

**Notes.**

a Different letters indicate significant differences with a *t*-Student test (*p* < 0.05) Percentages represent average values ± standard error.

### Expression of salicylic acid-associated genes

**Figure 5:**
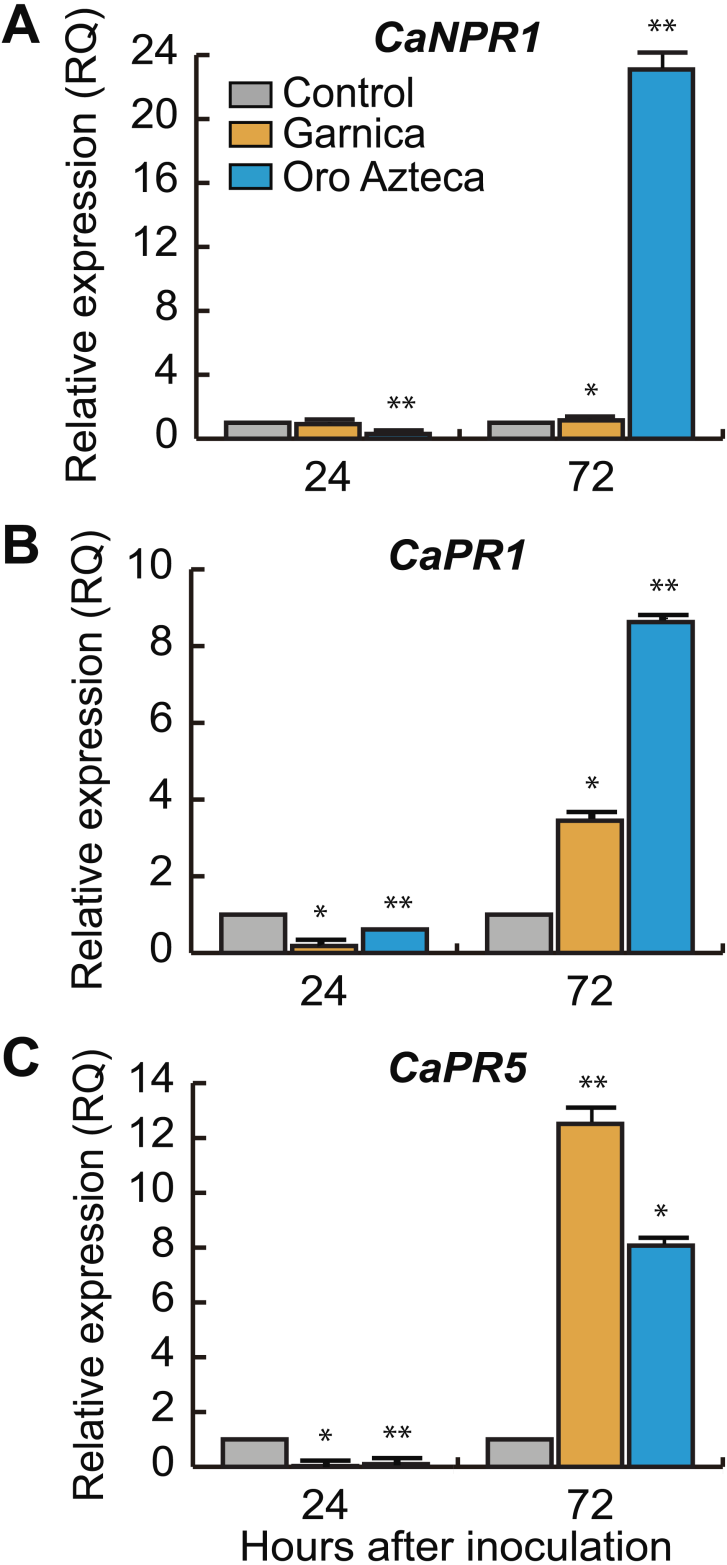
Relative expression of defense and salicylic acid-responsive genes. (A) *CaNPR1*, (B) *CaPR1* and (C) *CaPR5* relative expression at 24 and 72 hours after inoculation (hai). According to Tukey test (*p* < 0.05), one single asterisk indicates difference between the control and the inoculate disk of both cultivars, two asterisks mean differences with control and between cultivars.

Once we determined that the early recognition gene *CaNDR1b* was more highly expressed in cv. Oro Azteca leaf disks than in those of cv. Garnica at 72 hai, we analyzed the expression of some salicylic acid (SA) related genes. The transcriptional regulator of the SA-responsive gene *CaNPR1* was differentially expressed between cultivars (<0.0001, 7 d.f. at 24 and 72 hai), showing an expression up to 20-fold greater in cv. Oro Azteca as compared to the control and cv. Garnica leaf disks (Fig. 5A).

The SA-regulated gene *CaPR1* was about two-fold more highly expressed in Oro Azteca than in the Garnica infected leaf disks (Fig. 5B). Despite the thaumatin-like gene, *CaPR5* showed a greater expression level in the leaf disks from both cultivars than control, it was more highly expressed in the Garnica than in the Oro Azteca leaf disks at 72 hai (Fig. 5C).

## Discussion

### Incompatibility interaction between Oro Azteca and coffee leaf rust

Comparison of the proliferation of fungal structures in the infected leaf disks from Oro Azteca and Garnica varieties showed that despite similar percentages of spore germination and appressoria formation, the development of haustorial structures was significantly higher (*p* = 0.0001) in Garnica than in Oro Azteca leaf disks (Table 2 and Fig. 2); indicating that local responses of Oro Azteca leaves arrest fungal colonization before the haustorial stage. These results were corroborated by measuring the relation of gene abundance between the endogenous *H. vastatrix Hv40s-Rib* gene and *C. arabica CaUbiE2* (Fig. 3); this kind of evaluation on the host colonization has been described for some other pathogen models (Eshraghi et al., 2011).

Our results are consistent with those obtained by Martins & Moraes (1996), who evaluated the development of *H. vastatrix* race II on Mundo Novo, a susceptible cultivar, and Sarchimor, a resistant cultivar. Although the authors did not observe significant differences of spore germination and appressoria formation percentages between these two cultivars, they registered a haustorial development five-fold higher in cv. Mundo Novo as compared to the cv. Sarchimor segregants. Similarly, Ganesh et al. (2006) found that there was no haustorial formation during an incompatible interaction, considering this infection arrest as an indicator of resistance against races of *H. vastatrix*. The cytochemical characterization of the compatible interaction between cv. Caturra and *H. vastatrix* race III, performed by Silva et al. (1999), showed a similar proliferation of haustorial structures (64%) to that of the cv. Garnica leaf disks in this study at 120 hai (Table 2). Our results, consistent with previous studies, showed that defense mechanisms were activated before the formation of haustoria in the Mexican variety Oro Azteca. This arrest in the proliferation of *H. vastatrix* is correlated to the activation of genes related to the plant defense responses.

### Expression of the plant pathogen-recognition genes *CaNDR1* and *CaNBS-LRR*

We evaluated the expression of the previously reported non-race pathogen recognition gene *CaNDR1b* (Fernandez et al., 2004; Ganesh et al., 2006; Cacas et al., 2011). *CaNDR1b* has been identified within a group of genes that participate in the mechanisms of resistance to CLR (Fernandez et al., 2004; Cacas et al., 2011) at 24 hai. Several *NDR1* homologs have been identified in other plant species during incompatible plant-pathogen interactions (Century et al., 1997; Knepper, Savory & Day, 2011a; Lu et al., 2013; McNeece et al., 2017). *CaNDR1b* is a key gene in the activation of mechanisms of plant defense in response to pathogen-associated molecular patterns (PAMPs), to the pathogen-triggered immunity (PTI), and to the effector-triggered immunity (ETI) (Knepper, Savory & Day, 2011b). In the present study, *CaNDR1b* did not show overexpression at 24 hai. At 72 hai, however, its relative expression was at least 10-fold higher in Oro Azteca than in Garnica infected leaf disks (Fig. 4A), suggesting that *CaNDR1* could be involved in the recognition and defense responses to CLR in Oro Azteca cv. It would be interesting to determine in future studies its expression pattern between 24 and 72 hai, in order to compare the expression kinetics in Oro Azteca and Garnica cultivars.

Otherwise, the gene encoding the transmembrane protein pattern recognition receptor (PRR), *CaNBS-LRR*, showed an expression level below the control and susceptible Garnica in the cultivar Oro Azteca. This difference in expression was statistically significant at 24 and 72 hai (Fig. 4B). Members of this superfamily of receptor-like protein kinases are involved in the early recognition of ETI-activating pathogens, activating when pathogens deliver effectors that interfere with PTI and the pathogen infects the cells adjacent to the stomatal chamber (Jones & Dangl, 2006; Dodds & Rathjen, 2010; Knepper, Savory & Day, 2011b). This difference in the expression of this gene in Oro Azteca and Garnica is consistent with what we observed in fungal growth. Another reason for the difference could be that NBS-LRR proteins can be encoded by hundreds of genes in different plant species (McHale et al., 2006). Additionally, some other members of this gene family could be involved in the activation of this variety of Oro Azteca’s ETI, as opposed to only this specific gene whose expression we studied. Another hypothesis is that *CaNBS-LRR* expression induction could happen later than 72 hai, as previously reported by Florez et al. (2017).

### Expression of salicylic acid-associated genes

*CaNPR1* is identified as an expression regulator of pathogenesis-related proteins (PR) encoding genes and also as a response initiator associated with acquired systemic resistance (SAR) (Glazebrook, 2005; Barsalobres-Cavallari et al., 2013). Orthologs of this gene have shown the same function in other plant species, e.g., *Arabidopsis thaliana* (Shah, Tsui & Klessig, 1997), *Vitis vinifera* (Le Henanff et al., 2009); *Glycine max* (Sandhu et al., 2009) and *Gladiolus hybridus* (Zhong et al., 2015). In coffee, lower expression levels of *CaNPR1* have been reported in susceptible varieties (Barsalobres-Cavallari et al., 2013), with similar expression levels to those that we observed in the Garnica infected leaf disks, in contrast with the 20-fold induction in Oro Azteca (Fig. 5A). These results strongly suggest that resistance to CLR in Oro Azteca may be driven by the activation of the SAR mechanisms.

The behavior of the salicylic acid (SA)-induced gene *CaPR1* (Fig. 5B) was comparable to that which was reported by Ramiro et al. (2009), Diniz et al. (2012) and Florez et al. (2017); exhibiting a higher expression level in the resistant material than in the susceptible one. The protein family for which *CaPR1* encodes is related to fungal attacks (Jain & Khurana, 2018) and is used as a marker of a defensive state induced by SAR (van Loon, Rep & Pieterse, 2006). Hoegen et al. (2002) found that in potatoes infected with *Phytophthora infestans*, the highest concentration of *CaPR1* was in epidermal cells, stoma guard cells, glandular trichomes, and cells from the vascular system of the infected leaves. The lower number of *H. vastatrix* haustorial structures observed in cv. Oro Azteca in comparison to those observed in cv. Garnica could be related, at least partially, to this overexpression of gene *CaPR1*.

Finally, we evaluated the expression of the pathogen-induced gene *CaPR5* that encodes a thaumatin protein (Jain & Khurana, 2018). *CaPR5* showed an increased relative expression in both cultivars, Garnica and Oro Azteca, as compared with the control, but a higher induction in the cv. Garnica infected leaf disks (Fig. 4C). Studies performed in wheat varieties have shown higher expression levels in susceptible plants than in resistant materials at 24 and 72 hai with *Puccinia triticina* (Wang et al., 2010). The broad spectrum of stress-related signals that induce the expression of *CaPR5* in different plant models can explain these expression patterns (Wang et al., 2010).

## Conclusions

Altogether, our results suggest that defense mechanisms in Oro Azteca began with the early recognition of CLR by *CaNDR1*, activating the PTI. *CaNPR1*’s expression level could indicate the induction of SAR (Niu et al., 2016) through the SA signaling pathway. However, the way in which PTI and *CaNPR1* are correlated is not yet clear; whether the activation of the SA responsive genes in response to CLR in Oro Azteca could involve an HR should be explored in future studies.

##  Supplemental Information

10.7717/peerj.8345/supp-1 Data S1 Raw data: Fungal growth in cv. Garnica and Oro Azteca leaf-disks. Percentage of uredospore germination, appressorial and huastorial detection

10.7717/peerj.8345/supp-2 Data S2 Raw data: Expression of the *H. vastatrix* endogenous gene *Hv40s-Rib* relative to the coffee housekeeping gene* CaUbiE2.*

10.7717/peerj.8345/supp-3 Data S3 Raw data: Relative gene expression analysis of Garnica and Oro Azteca cultivars in response to *Hemileia vastatrix* infection

## Additional Information and Declarations

### Data Availability

The following information was supplied regarding data availability: Raw data are available as Supplemental Files.
